# Evaluation of the Impact of Anti‐C1q Autoantibodies on Cardiovascular Outcomes in Systemic Lupus Erythematosus

**DOI:** 10.1111/sji.70028

**Published:** 2025-04-21

**Authors:** Jessica S. Kleer, Andrea Kieninger‐Gräfitsch, Carlo Chizzolini, Uyen Huynh‐Do, Camillo Ribi, Marten Trendelenburg

**Affiliations:** ^1^ Laboratory of Clinical Immunology, Department of Biomedicine University of Basel Basel Switzerland; ^2^ Division of Internal Medicine University Hospital Basel Switzerland; ^3^ Department of Pathology and Immunology University Hospital Geneva Switzerland; ^4^ Department of Nephrology and Hypertension University Hospital Bern Switzerland; ^5^ Division of Immunology and Allergy, Department of Internal Medicine University Hospital Lausanne Switzerland

Dear Editor,

Complement C1q is implicated in pathogenic mechanisms of systemic lupus erythematosus (SLE). Beyond initiating the classical complement pathway, C1q has also several non‐canonical functions [1]. Our recent studies demonstrated a direct interaction between C1q and von Willebrand factor (vWF), particularly when C1q was bound to cholesterol crystals or deposited in atherosclerotic plaques, modulating macrophage responses and local inflammation [2, 3].

Anti‐C1q autoantibodies (anti‐C1q) are strongly associated with proliferative lupus nephritis (LN) and overall disease activity, and thus highly likely interfering with the functions of C1q [1]. Anti‐C1q target neo‐epitopes of the collagen‐like part of C1q including epitope A15 (formerly called ‘A08’) [4], that is also a binding site for vWF [5]. Considering the role of C1q in atherosclerosis [6], we hypothesise that the presence of anti‐C1q, particularly those targeting the A15 (and A09) epitope, influence the occurrence of cardiovascular disease (CVD) in SLE. We analysed data from 378 patients in the Swiss SLE Cohort Study (SSCS). Anti‐C1q and epitope‐specific antibodies (anti‐A09 and anti‐A15, respectively) were measured by ELISA [7]. CVD was assessed by the Systemic Lupus International Collaborating Clinics (SLICC) damage index [8], considering age at the first cardiovascular event, or, in patients without CVD, at the most recent SLICC evaluation. Traditional cardiovascular risk factors were included in the analysis as controls. Additionally, we analysed post‐thrombotic syndrome, a condition also characterised by an activation of coagulation.

Of the 378 SLE patients, information about the occurrence of CVD was available in 356. Of these patients, 59 had documented CVD, including cerebrovascular insult (*n* = 31), coronary artery disease (*n* = 27), myocardial infarction (*n* = 26), peripheral arterial disease (*n* = 13), mesenteric insufficiency (*n* = 1) and post‐thrombotic syndrome (*n* = 9).

Contrary to our hypothesis, we found no significant association between positivity for anti‐C1q (OR 0.42 [95% CI 0.16–1.09]), anti‐A15 (OR 1.35 [95% CI 0.75–2.41]), or anti‐A09 (OR 0.96 [95% CI 0.54–1.69]) and CVD. However, positive correlations were observed for ‘traditional’ risk factors such as age (OR 1.05 [95% CI 1.03–1.07]), antiphospholipid antibody (APL) positivity (OR 2.39 [95% CI 1.35–4.25]), arterial hypertension (OR 5.34 [95% CI 2.64–10.81]), history of smoking (OR 1.89 [95% CI 1.04–3.43]) and dyslipidemia (OR 14.98 [95% CI 3.35–67.03]) (Figure 1A). Anti‐A15 and anti‐A09 were excluded from multivariate logistic regression due to correlation with anti‐C1q [7], and dyslipidemia due to small sample size resulting in unstable estimates. After adjusting for confounders, the lack of association between anti‐C1q positivity and CVD remained, while positive correlations persisted for age (OR 1.05 [95% CI 1.02–1.08]), APL positivity (OR 2.51 [95% CI 1.10–5.74]) and arterial hypertension (OR 4.36 [95% CI 1.82–10.45]) (Figure 1B). Last, we could not detect associations between anti‐C1q positivity and specific CVD subcategories.

**FIGURE 1:**
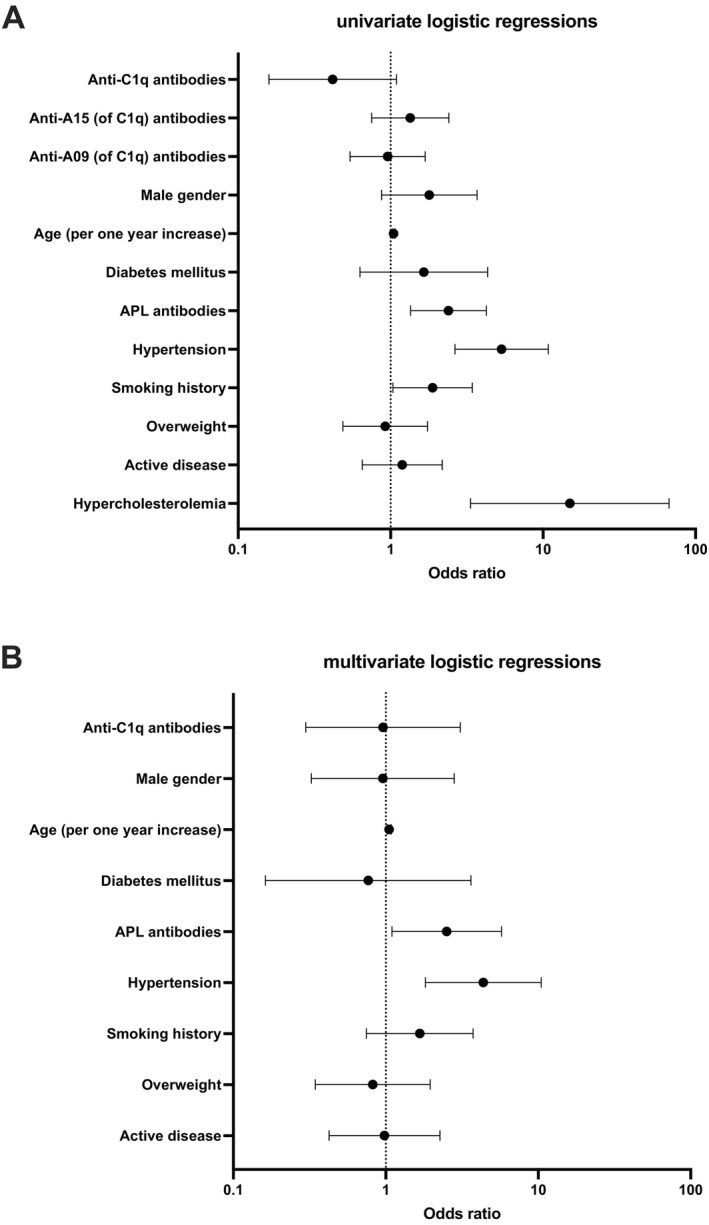
Logistic regression analysis of cardiovascular disease (CVD) risk factors. Part (A) displays odds ratios (ORs) and 95% confidence intervals (CIs) for the association between the occurrence of CVD and individual predictors in univariate logistic regression analyses. These predictors include anti‐C1q autoantibodies, epitope‐specific anti‐C1q antibodies targeting the A15 and A09 epitopes of C1q, and traditional cardiovascular risk factors. APL, antiphospholipid. Part (B) illustrates the ORs and 95% CIs for predictors included in the multivariate model assessing the relationship between various factors and the occurrence of CVD. Odds ratios are plotted on a logarithmic scale, with dashed lines indicating a reference OR of 1 (no association).

Although anti‐C1q is a known marker for SLE disease activity [1], our data do not support associations between anti‐C1q or their N‐terminal‐specific forms (anti‐A15 and anti‐A09) and the occurrence of CVD in SLE patients. Potential limitations include that SSCS does not primarily capture detailed cardiovascular outcomes, and a single anti‐C1q measurement possibly misses subtle associations. Nonetheless, we confirmed the well‐established APL–CVD link in a large, prospective cohort.

Thus, our analyses do not support the hypothesis of anti‐C1q affecting the occurrence of CVD in SLE patients, but longitudinal studies may be necessary to fully exclude such an association.

## Ethics Statement

The studies involving human participants were reviewed and approved by Swissethics (ethical committee of the Canton Vaud, Switzerland Ref. No. 2017‐01434). The studies were conducted in accordance with the local legislation and institutional requirements. The participants provided their written informed consent to participate in this study.

## Conflicts of Interest

The authors declare no conflicts of interest.

## Data Availability

The raw data supporting the conclusions of this article will be made available by the authors, without undue reservation.
